# Supplementary material for: Threshold-based exploitation of noisy label in black-box unsupervised domain adaptation
Source: PLoS One. 2025 May 12;20(5):e0321987. doi: 10.1371/journal.pone.0321987 (PMC12068613; doi:10.1371/journal.pone.0321987)
Supplement: Attachment — Submitted filename: response to reviewers.pdf [file pone.0321987.s001.pdf]

Threshold-Based Exploitation of Noisy Label in Black-box Unsupervised Domain Adaptation

Huiwen Xu, Jaeri Lee, and U Kang

We thank the reviewers for the high-quality comments. We address the comments as follows.

Reviewer 1

R1.1 Proofread the entire manuscript.

A1.1 The revised manuscript reflects these changes to improve readability and clarity.

R1.2 Draw a graphical abstract of your proposed approach.

A1.2 The overall structure of our proposed approach is depicted in Fig. 1. During the division phase, we ensure the selection of data with relatively less noise for distillation. In the training phase, we utilize the already divided clean data along with noisy data with high confidence for domain adaptation, reducing information loss in the process. Additionally, we leverage regularizations to exploit the structure information of the target data.

R1.3 Compare your approach with previous approaches.

A1.3 We provide a more detailed comparison of our approach with previous approaches in the second paragraph of the "Related Works" section.

R1.4 Explain about features you are using in this study.

A1.4 Details about the features used in our study are provided in the "Proposed Method" section. Our approach tackles three key challenges: dividing target data into clean and noisy subsets using adaptive thresholds to handle hard-to-learn classes, extracting meaningful insights from noisy labels through negative learning to mitigate performance degradation, and capturing the structural information of the target data using entropy and consistency regularization. These strategies enable better utilization of noisy labels and improved learning of the target domain's intrinsic patterns for robust model performance.

Reviewer 2

R2.1 There should be a deeper discussion of hyperparameter sensitivity, particularly the thresholds and regularization parameters critical to TEN's performance.

A2.1 We expanded the discussion in the "Hyperparameter Sensitivity (Q5)" subsection of the "Experiments" section.

R2.2 While the computational efficiency of the method is implied, explicit comparisons of overhead against baselines would strengthen the practical appeal.

A2.2 While computational efficiency is important, our primary contribution is the significant boost in accuracy, demonstrating the practical potential of our approach. We added the discussion in the last paragraph of "Conclusion" section.

R2.3 The authors should discuss the potential failure cases or generalizability to domains beyond image classification limits the broader applicability of the findings.

A2.3 We added the discussion in the last paragraph of "Conclusion" section.

R2.4 The authors should include references [1, 2, 3] related to UDA and black-box UDA in related work.

A2.4 We incorporated the suggested references into the second paragraph of "Related Works" section.

References

[1] Zhang J, Huang J, Jiang X, Lu S. Black-box Unsupervised Domain Adaptation with Bi-directional Atkinson-Shiffrin Memory. In: IEEE/CVF International Conference on Computer Vision, ICCV 2023, Paris, France, October 1-6, 2023;.

[2] Zhu C, Zhang L, Luo W, Jiang G, Wang Q. Tensorial multiview low-rank high-order graph learning for context-enhanced domain adaptation. Neural Networks. 2025;181:106859.

[3] Zhu C, Wang Q, Xie Y, Xu S. Multiview latent space learning with progressively fine-tuned deep features for unsupervised domain adaptation. Inf Sci. 2024;662:120223.
